# Effect of maternal sleep on embryonic development

**DOI:** 10.1038/s41598-022-21516-6

**Published:** 2022-10-12

**Authors:** Alexander Vietheer, Torvid Kiserud, Øystein Ariansen Haaland, Rolv Terje Lie, Jörg Kessler

**Affiliations:** 1 Department of Obstetrics and Gynecology, Haukeland University Hospital, Jonas Lies vei 72, 5053 Bergen, Norway; 2 Department of Clinical Science, Neonatal Research Group Western Norway, Maternal Fetal, University of Bergen, Bergen, Norway; 3 Department of Global Public Health and Primary Care, University of Bergen, Bergen, Norway; 4 Centre for Fertility and Health, Norwegian Institute of Public Health, Oslo, Norway

**Keywords:** Physiology, Reproductive biology, Developmental biology, Embryogenesis, Embryology, Intrauterine growth, Lifestyle modification, Preventive medicine, Ultrasonography, Disease prevention, Medical imaging

## Abstract

The concept of developmental origin of health and disease has ignited a search for mechanisms and health factors influencing normal intrauterine development. Sleep is a basic health factor with substantial individual variation, but its implication for early prenatal development remains unclear. During the embryonic period, the yolk sac is involved in embryonic nutrition, growth, hematopoiesis, and likely in fetal programming. Maternal body measures seem to influence its size in human female embryos. In this prospective, longitudinal observational study of 190 healthy women recruited before natural conception, we assessed the effect of prepregnant sleep duration (actigraphy) on the fetal crown-rump-length (CRL) and yolk sac size (ultrasound). All women gave birth to a live child. The prepregnancy daily sleep duration had an effect on the male yolk sac and CRL at the earliest measurement only (7 weeks). I.e., the yolk sac diameter decreased with increasing sleep duration (0.22 mm·h^−1^d^−1^, 95%CI [0.35-0.09], *P* < *0.01*), and CRL decreased (0.92 mm·h^−1^d^−1^, 95%CI [1.77-0.08], *P* = 0.03). Since there was no association at the second measurement (10 weeks), and in the group of female fetuses at any measure point, we suggest a sex- and time-dependent embryonic adaptation to sleep generated differences in the intrauterine environment in normal pregnancies.

## Introduction

It is widely accepted that sleep is essential for our health and physiologic homeostasis. This is certainly the case for pregnancy, as sleep disturbances are associated with pregnancy complications^1^, such as preeclampsia, gestational diabetes, preterm birth, and prolonged birth duration^2–6^. Ethical reasons restrict in vivo experiments, so studies of sleep effects on human embryonic physiology and development are scarce^1,7^. This is a dilemma, since physiology and its normal variation lay the foundation for the understanding of pathology and disease. Why is, for example, more than one nightshift per week associated with a 30% higher risk of early pregnancy loss?^8^ What are the pathophysiologic mechanisms? And, how much of regular sleep is needed in pregnancy?

In vitro experiments in assisted reproduction and animal studies demonstrate a possible circadian rhythm link to embryonic development and epigenetic programming via melatonin, cortisol, and sex steroids^9–17^. Further, deep sleep influences the growth hormone secretion in humans^18^, which in turn has notable effects on fertility and the intra-uterine environment^19^. Likewise, sleep disturbances (such as insomnia) are linked to HPA-axis activation^20^ and rise in cortisol levels^21^—a stress response with capability to impair fertility during critical periods, ovulation, implantation, and placental growth and development^22^. Nevertheless, animal studies, in vitro studies, and studies on infertile cohorts seeking health services cannot necessarily reflect the normal physiology of healthy women; this is where we can employ ultrasound as a non-invasive method.

In pregnancy, the human yolk sac is one of the first structures that can be assessed by ultrasound^23^. It has a complex surface structure that ensures embryonal nutrition and growth before the placenta is sufficiently developed^24,25^, and its size is associated with fetal anomalies and unfavorable pregnancy outcomes^26–28^. Previous animal studies have demonstrated yolk sac size variation related to environmental factors, e.g., noise, temperature, and nutrition^27,29,30^. Further, women’s body composition, i.e., lower weight and height have been linked to larger yolk sac^31^, while human sleep with its large-scale physiologic variation has not been a focus of research^32,33^.

Thus, we hypothesized that maternal sleep has an effect on embryonic development in humans, and aimed at assessing the effect of maternal sleep recordings on measurements of yolk sac size and crown-rump-length (CRL).

## Method

The present study is a prospective, longitudinal observational study of the relation of actigraphy measured sleep in healthy women with ultrasound assessed early fetal development. It is embedded in the ongoing CONIMPREG research program which collects data of healthy women from before conception, through pregnancy, and until the child reaches the age of 5 years.

### Study cohort

**Figure 1:**
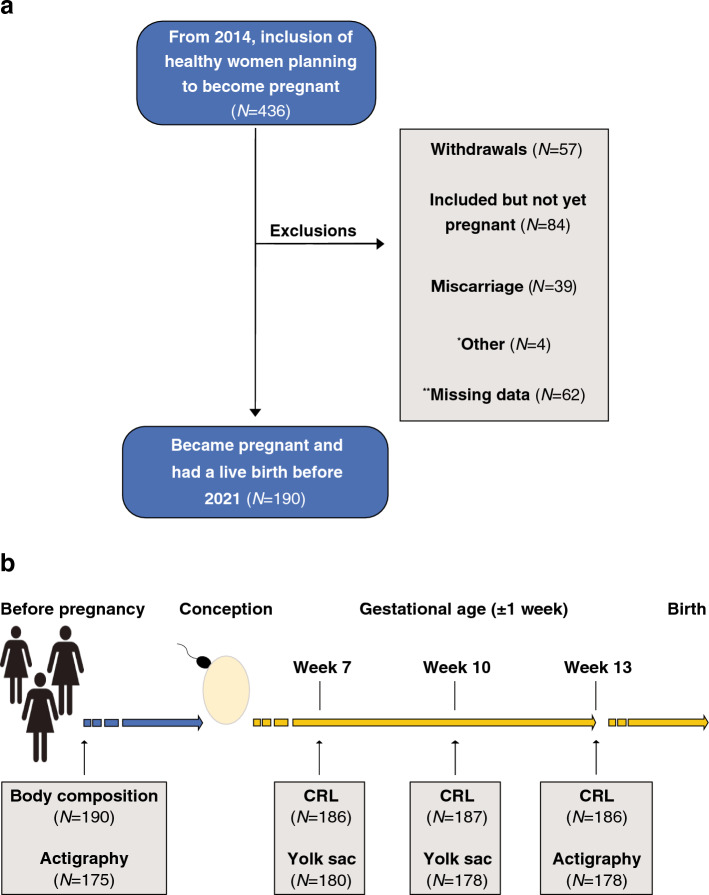
(**a**) Flowchart of the study population with the participant exclusions—*other: 1 twin pregnancy, 2 abortions > 18 weeks (fetal anomaly), and 1 irregular menstrual cycle; **missing data: Pregnancies with incomplete entry data (*N* = 54) and pregnant < 13 weeks at time of data extraction (*N* = 8). (**b**) Timing of measurements and recordings.

The participants were recruited during the period 2014–2020 by means of social media (targeted Facebook® advertisements) and posters. Healthy non-smoking women aged 20–35 years with a BMI of 18–30 kg/m^2^ were eligible provided they had an uncomplicated obstetric history and no chronic diseases or fertility problems. The participants did not use contraceptives at study entry, including the preceding month. The participants who did not conceive during any of the 6 monitored cycles allotted to them, discontinued the participation (Fig. 1). The study was approved by the Regional Committee for Medical Research Ethics South East Norway (REK South East, ref. 2013/856a). Written informed consent was obtained from all participants and all research was performed in accordance with relevant guidelines and regulations.

### Data collection

The current study includes data from four consecutive study visits (Fig. 1). At the first visit (i.e., at inclusion and before the participants attempted to conceive), prepregnant height was measured manually using a wall-mounted stadiometer^34^ and weight was measured digitally using hand-to-foot bioelectrical impedance analysis (model BC-418, Tanita, Tokyo, Japan). The body fat percentage was estimated using the instrument’s computer software, and the lean body mass was calculated by subtracting the body fat mass from the total body weight. Measurements were performed as recommended by the manufacturer^35^ and immediately followed by actigraphy recording. Following inclusion, the woman monitored her menstrual cycles by recording events, such as bleeding, sexual intercourse, and pregnancy tests. Once she had a positive pregnancy test, she reported to the study office and was scheduled for the second visit at gestational age (GA) 7 ± 1 weeks calculated from her last menstrual period (LMP). At that second visit, the gestational age was confirmed using transvaginal ultrasound scanning. The embryonic CRL and secondary yolk sac were assessed^36^. Later in pregnancy, at the third visit, 10 ± 1 weeks, the ultrasound measurements were repeated and at the fourth visit, 13 ± 1 weeks (end of the 1st trimester), maternal body composition and sleep duration were re-assessed along with the sonographic measurement of the CRL (Fig. 1).

### Actigraphy

Sleep duration was recorded with the SenseWear Mini Armband, model MF-SW, BodyMedia, Pittsburgh, PA, USA. The monitor was worn on the upper posterior aspect of the nondominant arm for 4 days^37^ and the recording started at midnight. The SenseWear actigraph is a wireless, noninvasive monitor with a sampling rate of 32/sec and incorporates triaxial accelerometry, heat flux, galvanic skin response, skin temperature, and near-body temperature, and takes into account information on sex, age, height, and weight^38^. Sleep and wakefulness are discriminated based on motion and skin temperature in 1-min epochs. Sleep raw data were processed and summarized using the manufacturer’s analysis software (SenseWear Professional, version 8.0.0.2903, Body Media) and exported into Excel (Microsoft Office, Excel version 2016, Redmond, WA, USA). This actigraph has been compared with other models and standard measurement methods in clinical and experimental settings^39–44^. Recently, our group used this monitor to measure sleep in pregnancy with results in good agreement with other actigraphy studies^33^. The sleep efficiency (SE) was calculated by the software as sleep proportion of the time lying down.

### Embryonic and fetal assessment

The fetal crown-rump-length and yolk sac size were examined by seven obstetricians and one certified midwife using a transvaginal ultrasound transducer, 6–12 MHz, Voluson Expert E8; (GE Medical Systems, Kretz Ultrasound, Zipf, Austria). The yolk sac diameter was determined as the average of two perpendicular diameters repeated thrice^31^. Yolk sac growth rate was calculated as the difference in yolk sac diameter divided by the number of days between the two measurements.

### Statistics

Statistical analysis was performed using SPSS (version 24, Armonk, NY, USA), R (Foundation for Statistical Computing, version 4.1, Vienna, Austria), and R-studio (Integrated development for R, Boston, MA, USA) software. Sampling days were excluded from the statistical analyses when the data loss exceeded 6% of a single day. Mean, standard deviation (SD) with minimum and maximum value were calculated for each continuous variable, and frequencies and proportions were calculated for categorical variables. When the distribution was asymmetric the median and interquartile range were reported. In addition, the 95% confidence intervals (CIs) of the mean were calculated for sleep parameters, the number of recorded days, the frequency of weekend days, and the CRL and yolk sac size with their GA at the measurements.

Linearity assumptions and normal distribution of the residuals were ascertained, and ordinary least square linear and quantile regression models were used to analyze the yolk sac size association with the sleep duration before pregnancy and the sleep duration at the end of the first trimester (week 13). Results were compared with results from robust regression methods (iterated reweighted least squares by Huber weights and bi-square weighting) and heteroskedastic methods (sandwich variance estimators). Calculations were carried out with and without model outliers (Cooks distance > 4 times). Mixed models were used for the repeated measurements analysis. The regression models were calculated with and without fetal sex stratification and stratification from lower to higher quartiles of the sleep duration. In sub analysis, we controlled for GA, maternal age, parity, physical activity level before conception, and maternal body composition parameters (i.e., height, weight, body mass index, lean body mass, and body fat percent). Those factors were added one by one to the primary model (yolk sac or CRL size by total sleep duration) and were included if they were altering the effect-size of the association notably. As measures of fit, the adjusted *R*–squared and Akaike information criterion were calculated. Differences between the regression models were tested using analysis of variance methods. The different strata were also compared with paired and unpaired Student’s *t*-test or nonparametric tests.

### Ethical declaration and informed consent

The study was approved by: Regional Comittee for Medical Research Ethics South East Norway (REK South East, ref. 2013/856a). E-mail: rek-sorost@medisin.uio.no. Written informed consent was obtained from all participants and all research was performed in accordance with relevant guidelines and regulations.

## Results

Of 436 eligible participants, 190 (43.6%) conceived with a successful pregnancy and provided sufficient data to be included in the present study (Fig. 1). Their demographic information and obstetric outcomes are provided in Tables 1 and 2.

**Table 1:** Descriptive statistics of the participants—missing values (Nmiss); mean; standard deviation (SD); range (Min, Max). Training efforts established using a non-validated questionnaire that each participant completed at study entry.

*N* = 190	Frequency	Nmiss	Mean	SD	Min	Max
Age (years)		None	29.0	3.1	20.0	35.0
Height (cm)		None	167.7	6.2	149.0	185.0
Weight (kg)		None	64.7	8.3	47.1	89.8
BMI		None	23.0	2.6	17.8	29.9
Lean body mass (kg)		None	45.7	3.8	36.0	55.6
Body fat (%)		None	28.8	5.5	15.9	41.9
Cycle length (days)		None	28.5	1.7	24	35
Parity		None				
0	89 (46.8%)					
1	79 (41.6%)					
≥ 2	22 (11.6%)					
Training efforts		None				
None	3 (1.6%)					
Effortless walk	46 (24.2%)					
< 3 times·week^-1^	90 (47.4%)					
≥ 3 times·week^-1^	51 (26.8%)

**Table 2:** Selected descriptive statistics of pregnancy, common gestational diseases (i.e., hypertension, preeclampsia (PE), diabetes), and newborns—missing values (Nmiss); mean; standard deviation (SD); range (Min, Max).

*N* = 190	Frequency	Nmiss	Mean	SD	Min	Max
Hypertension| PE	6 (3.2%)	None				
Gestational diabetes	7 (3.7%)	None				
Pregnancy length (days)		None	276.7	10.7	214	297
Preterm birth	6 (3.2%)	None				
Birthweight (g)		None	3566	491	1230	4910
Neonatal Sex (female)	94 (49.5%)	None				
Apgar < 7 (5 min)	2 (1.1%)	1				

**Figure 2:**
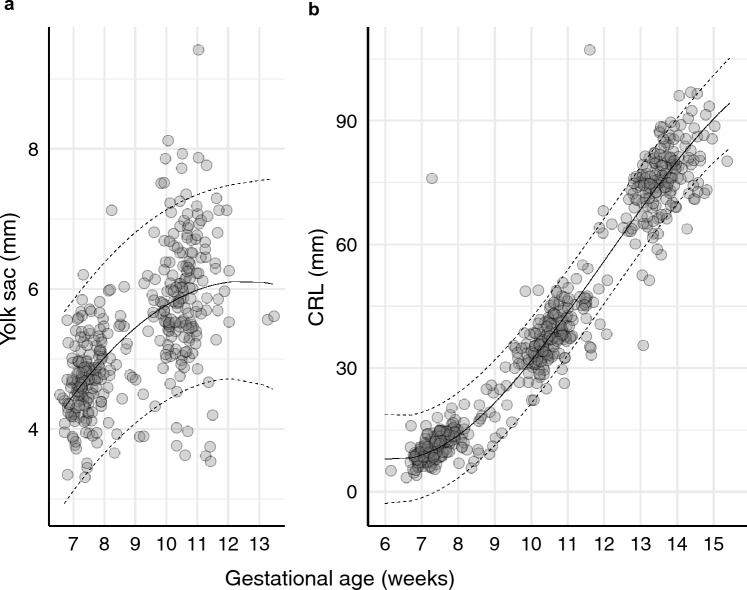
(**a**) First and second yolk sac measurements (total *N* = 358), and (**b**) the three serial crown-rump-length (CRL) measurements (total *N* = 559), presented with predicted mean (mixed model with random intercept) and 95% prediction band. Gestational age was based on last menstrual period.

During actigraphy, 92.1% of all days before conception and 93.7% at the end of the first trimester were successfully recorded. The yolk sac was measured totally 358 times, and the CRL 559 times (Fig. 2). The summary statistics of the actigraphy recording, yolk sac diameter, and CRL are shown in Tables 3 and 4.

**Table 3:** Summary statistics of the actigraphy data from 190 low-risk pregnancies before pregnancy and at 13 gestational weeks—number of measurements (*N*); mean; standard deviation (SD); 95% confidence interval (95%CI). The sleep difference was calculated from the two sleep measurements; time to conception was calculated as number of days from actigraphy recording at inclusion to day 14 of the menstrual cycle when the woman conceived.

Term	*N*	Mean	SD	95%CI
Sleep before pregnancy: Days before conception	178	51.7	53.1	(43.8–59.5)
Sleep before pregnancy: Number of recorded days	177	3.7	0.7	(3.6–3.8)
Sleep before pregnancy: Daily sleep time (min)	175	423.1	54.1	(415.0–431.2)
Sleep efficiency before pregnancy (%)	175	83.0	6.3	(82.0–83.9)
Week 13: GA at 2nd sleep recording (weeks)	181	13.2	0.8	(13.1–13.3)
Week 13: Sleep measurement (recorded days)	178	3.7	0.6	(3.6–3.8)
Week 13: Daily sleep time (min)	178	460.5	66.6	(450.6–470.3)
Week 13: Sleep efficiency (%)	178	81.6	7.0	(80.6–82.7)
Week 13–prepregnancy: Sleep difference (min)	172	37.9	61.7	(28.6–47.2)

**Table 4:** Summary statistics of the ultrasound data with number of measurements (*N*), mean, standard deviation (SD), and 95% confidence interval (95%CI) from 190 low-risk pregnancies—gestational age by LMP (GA) at the measurement; yolk sac size; crown-rump-length (CRL).

Term	*N*	Mean	SD	95% CI
Week 7: 1st measurement; GA (weeks)	186	7.5	0.9	(7.4–7.7)
Week 7: 1st crown-rump-length (mm)	186	11.4	3.8	(10.8–11.9)
Week 7: 1st yolk sac diameter (mm)	180	4.7	0.6	(4.7–4.8)
Week 10: 2nd measurement; GA (weeks)	187	10.6	0.9	(10.5–10.7)
Week 10: 2nd crown-rump-length (mm)	187	37.8	6.9	(36.8–38.8)
Week 10: 2nd yolk sac diameter (mm)	178	5.9	0.9	(5.8–6.1)
Week 7–10: Yolk sac growth rate (mm·week^-1^)	170	0.038	0.033	(0.033–0.043)
Week 13: 3rd measurement; GA (weeks)	185	13.6	1.0	(13.4–13.7)
Week 13: 3rd crown-rump-length (mm)	186	76.4	8.1	(75.2–77.6)

The median time from the first sleep recording to estimated day of conception (i.e., 14 days after LMP) was 36 days (IQR 10–75). The majority conceived within the two first menstrual cycles. The total sleep duration before conception was 38 min shorter than at the end of the first trimester (95%CI [28.6–47.2], *P* < *0.01*), the total length of actigraphy and the frequency of recorded weekend days did not differ before and after conception.

Prepregnant sleep duration, but not the other sleep parameters before pregnancy had an effect on the yolk sac size (Supplementary Table S1). Generally, the effect on the yolk sac and CRL was weaker when the relation to measurement time was not taken into account (Supplementary Tables S1 and S2). The following analyses were stratified, all grouped according to time of the measurement at week 7, week 10, and week 13 for the CRL (Table 5 and 6).

**Table 5:** Prediction of the yolk sac diameter (all, males, and females) at 7 weeks and 10 weeks of gestation by total daily sleep duration before pregnancy and at 13 weeks of gestation. Calculated using ordinary least square regression models—unstandardized regression coefficient (Effect); adjusted R squared (Adj.R2); 95% confidence interval (95%CI); AIC (Akaike information criterion).

Group	*N*	Effect	95% CI	Adj.*r*2	AIC	*p*
**Yolk sac at week 7 by sleep duration before pregnancy**						
All	165	−0.12 mm·h^−1^·d^−1^	(−0.22 to −0.03)	0.03	291.4	**0.01**
Male	84	−0.22 mm·h^−1^·d^−1^	(−0.34 to −0.09)	0.11	133.6	** < 0.01**
Female	81	−0.04 mm·h^−1^·d^−1^	(−0.19–0.10)	−0.01	157.4	0.53
**Yolk sac at week 7 by sleep duration at the end of first trimester**						
All	168	−0.06 mm·h^−1^·d^−1^	(−0.14–0.02)	0.012	300.3	0.15
Male	84	−0.13 mm·h^−1^·d^−1^	(−0.25 to −0.01)	0.056	142.1	**0.03**
Female	83	−0.01 mm·h^−1^·d^−1^	(−0.12–0.09)	0.001	159.9	0.79
**Yolk sac at week 10 by sleep duration before pregnancy**						
All	164	−0.07 mm·h^−1^·d^−1^	(−0.22–0.09)	0.004	444.3	0.40
Male	82	−0.07 mm·h^−1^·d^−1^	(−0.29–0.14)	0.005	213.6	0.51
Female	81	−0.06 mm·h^−1^·d^−1^	(−0.29–0.14)	0.004	234.9	0.59
**Yolk sac at week 10 by sleep duration at the end of first trimester**						
All	167	−0.04 mm·h^−1^·d^−1^	(−0.17–0.08)	0.003	450.7	0.48
Male	83	−0.10 mm·h^−1^·d^−1^	(−0.28–0.09)	0.013	215.8	0.29
Female	83	−0.01 mm·h^−1^·d^−1^	(−0.18–0.17)	0.000	238.8	0.79

Significant are in bold.

**Table 6:** Prediction of the fetal crown-rump-length (CRL) measured at gestational week 7, 10, and 13 (all, males, and females) by total daily sleep duration before pregnancy and at gestational week 13. Calculated using ordinary least square regression models—unstandardized regression coefficient (Effect); adjusted R squared (Adj.R2); 95% confidence interval (95%CI); AIC (Akaike information criterion).

Group	*N*	Effect	95% CI	Adj.*r*2	AIC	*p*
**CRL at week 7 by sleep duration before pregnancy**						
All	171	−0.69 mm·h^−1^·d^−1^	(−1.32 to −0.05)	0.02	950.9	**0.03**
Male	87	−0.92 mm·h^−1^·d^−1^	(−1.77 to −0.08)	0.04	472.5	**0.03**
Female	83	−0.53 mm·h^−1^·d^−1^	(−1.48–0.42)	0.002	478.9	0.27
**CRL at week 10 by sleep duration before pregnancy**						
All	173	−0.44 mm·h^−1^·d^−1^	(−1.57–0.70)	−0.002	1163.8	0.45
Male	88	−0.30 mm·h^−1^·d^−1^	(−1.82–1.21)	−0.009	579.8	0.69
Female	84	−0.58 mm·h^−1^·d^−1^	(−2.31–1.15)	−0.006	586.6	0.51
**CRL at week 13 by sleep duration before pregnancy**						
All	164	0.02 mm·h^−1^·d^−1^	(−1.33–1.36)	−0.005	1205.1	0.98
Male	82	0.39 mm·h^−1^·d^−1^	(−1.60–2.38)	−0.010	611.4	0.70
Female	81	−0.23 mm·h^−1^·d^−1^	(−2.09–1.63)	−0.01	598.3	0.81

Significant are in bold.

### Relation between daily sleep duration and yolk sac size

**Figure 3:**
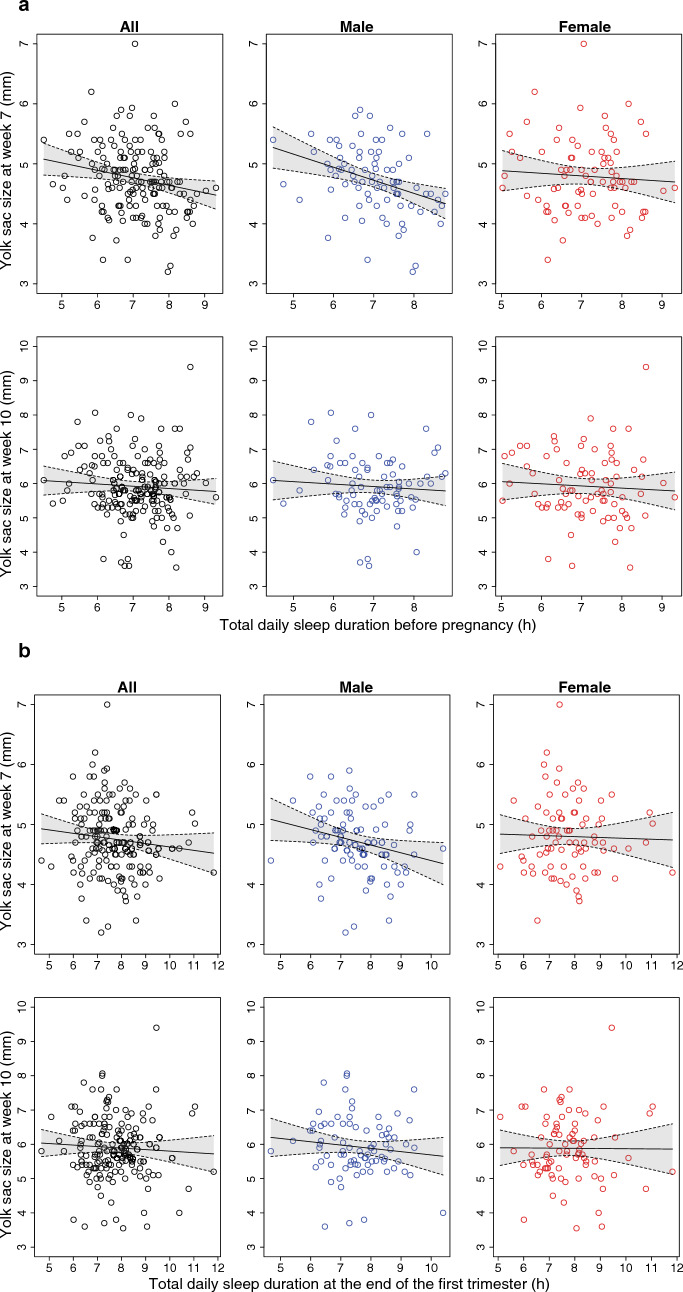
Effect of daily total sleeping time before pregnancy (**a**), and effect of the daily total sleeping time at 13 weeks (**b**) on the first (upper row) and second yolk sac measurement (lower row) in naturally conceived healthy pregnancies presented with regression line and its 95% confidence interval. The first column represents the total dataset and the second and third the analysis according to embryonic sex.

At the first ultrasound measurement (week 7), but not at the second (week 10), we found a sex-dependent negative effect of the prepregnant sleep duration on the yolk sac of male embryos (Fig. 3 and Table 5), i.e., longer sleep before pregnancy was associated with smaller yolk sac sizes.

It was noted that the sleep duration recorded later, at the end of the first trimester (week 13), was similarly linked to the yolk sac size at 7 weeks, but here, the effect was weaker (Fig. 3 and Table 5).

There was a considerable variation in the yolk sac growth rate between the two measurement time points (Supplementary Fig. S1)*,* but no association between the average growth rate and sleep duration (Supplementary Table S3).

**Figure 4:**
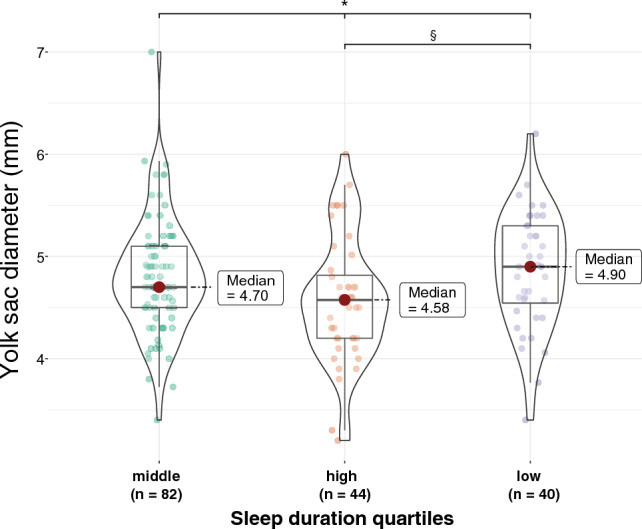
Raw-data-descriptive-inferential-statistics plot of the yolk sac size at week 7 (N = 166) grouped according to prepregnant sleep duration quartiles: Low sleep duration range (4 h 30 min–6 h 28 min); middle sleep duration range (6 h 29 min–7 h 40 min); high sleep duration range (7 h 42 min–9 h 19 min)—^*^ Kruskal–Wallis test (*p* = 0.03; ^§^ Post-hoc-test (Dunn’s test), *p* = 0.038.

Grouping of the sleep duration in quartiles, however, suggested that the yolk sac size was larger for the lowest sleep quartile than for the highest (Fig. 4); for these quartiles—lowest and highest—the sleep-yolk sac association also tended to be stronger.

Further, the relation between sleep duration before pregnancy and the embryonic yolk sac at week 7 was consistent across the yolk sac deciles (Supplementary Figs. S2 and S3)*,* and still present after adjustment for maternal characteristics and GA (Supplementary Tables S4 and S5). In sub-analyses, these adjustments were made for all models (i.e., yolk sac at 7 and 10 weeks), with preconception sleep duration and sleep duration at the end of the 1st trimester (13 weeks) as predictor, all stratified and unstratified by fetal sex. In none of the models these adjustments changed our conclusions.

### Maternal sleep duration and embryo size through the first trimester

Similar to the association between yolk sac diameter and sleep duration, the embryonic CRL was shorter when the prepregnant sleep duration increased. Stratification by the time of measurement and fetal sex revealed a comparable pattern, i.e., the effect was confined to male embryos at week 7, not later (week 10 or week 13) (Table 6). The sleep duration at the end of the 1st trimester (week 13) was not associated with any of the CRL measurements (males or females) at week 7, week 10, or week 13. Adjustments for maternal age, parity and body composition did not change these results significantly.

Finally, the interval from actigraphy sleep measurement before pregnancy to LMP/estimated conception was neither influencing the CRL-sleep-association nor the yolk sac-sleep-association (i.e., the number of menstrual cycles to achieve pregnancy was irrelevant for these relations).

## Discussion

This study demonstrates a sex- and time-dependent relation between prepregnant sleep duration and human embryonic development. The association between a shorter maternal sleep duration before pregnancy and a larger yolk sac and CRL was confined to male embryos at 7 weeks of gestation.

However, this effect on 7-weeks’ embryos could be traced later in pregnancy. I.e., although sleep duration assessed at 13 weeks was 38 min longer, it still had a distributional pattern that significantly linked it to the yolk sac size at 7 weeks.

A strength of the study is the prospective longitudinal design to address our study questions. Secondly, our participants were healthy women who conceived naturally without any influence of hormonal treatment commonly used in assisted reproduction. However, our study did not discriminate daytime and nocturnal sleep duration. The total daily sleep duration included daytime naps and does not necessarily reflect the sleep quality and quantity at night. In addition, we cannot be certain that the sleep patterns recorded before pregnancy, although close to conception (Table 2), were continued into early pregnancy. An additional use of sleep diaries would have strengthened the study and provided a deeper insight. On the other hand, the fact that the sleep duration pattern at 13 weeks was similarly related to yolk sac and embryo at 7 weeks as was prepregnant sleep duration, suggests that the sleep pattern was similarly distributed through the entire period.

Stress, physical and psychological, are known confounders with quite similar hormonal responses, but the present study was not designed to disentangle such factors from sleep effects. The study population, however, consisted of healthy women with no history of chronic diseases or risk factors, and the chance for being under chronic physical and psychological stress should therefore be low.

There is some evidence from animal studies supporting an effect of environmental factors on the yolk sac (e.g. temperature, nutrition, and noise)^29,30,45,46^, but extrapolation to human conditions may not be warranted as yolk sac development and implantation mechanisms differ between species^25,47,48^. However, a human study found that the yolk sac diameter was influenced by maternal body size^31^. They reported a smaller yolk sac at 8–12 weeks when maternal prepregnant height or weight was high. The relation was sex-dependent, i.e., shown only in female fetuses. Conversely, adjustment for maternal body composition parameters, including height and weight, did not change our estimates of the relation between daily sleep duration and yolk sac size (Supplementary Tables S4 and S5). We suggest to consider this rather as a concept than contradiction. The first trimester is a developmental rush hour. From conception and implantation to the end of week 13, myriads of developmental steps are taking place with different time windows opening and closing at high frequency. In the current study the sleep effect on the yolk sac was present at week 7, but not 3 weeks later supporting this notion.

Our findings can be explained by at least two different biological models. First, the increase of the yolk sac size could compensate for a less favorable intrauterine environment due to a shorter sleep duration. This concept has been suggested before (in connection with maternal body size) and implies that the human yolk sac can compensate for reduced access to maternal nutrients by means of a larger surface^31^. It is in line with the time-dependent and early effect on the yolk sac at 7 weeks: early nutrition depends foremost on the yolk sac; later, there is a gradual transition to a hemotrophic placental supply^24,25^. The similar association of the embryonic CRL at week 7 (Table 6) may appear inconsistent with such a model of yolk sac compensation, but could be explained by a delay of the compensatory effect.

A second model would be that there is a systematic shift of the embryonic or fetal age related to the measured sleep duration. I.e., both time of ovulation with the fertile window (day 8–15)^49^ and time of implantation vary (i.e., day 6–12 after ovulation)^50^. The influence of sleep on the HPA-axis, estradiol, melatonin, and other hormones^20,51^ can cause menstrual cycle changes^11,18,20,21,51–54^ and earlier ovulation. In addition, the interval from ovulation to implantation is inversely correlated with the first trimester measurement of the fetal CRL^55^. Thus, an association between the prepregnant sleep duration with early ovulation or shorter ovulation–implantation interval would result in a systematically larger yolk sac diameter and fetal CRL at any estimated GA by the LMP-method. This theory provides an explanation why both the yolk sac size and the CRL are sleep-related in early gestation. Later when size variation increases, due to individual embryonic and yolk sac growth or because the yolk sac dissolves^25,56^, these sleep-related findings may not be traceable.

The present study also demonstrated a fetal sex-dependent sleep-yolk sac association that was present only for male embryos. Weinberg et al. reported already in 1995 that women with a short follicular phase before conception tended to give birth to males^57^. It is tempting to propose that a similar mechanism could explain the present results. Nevertheless, sexual dimorphism alone could explain sex-specific environmental effects including a sex-specific plasticity that depends on embryonic age^58^. That means, time windows for developmental influence may differ between the sexes as seen in the present and a previous study on yolk sac size^31^. Male embryos at 7 weeks in the present study were sensitive to maternal sleep, while female embryos after 8 weeks in the previous study were sensitive to maternal body height and weight, which could be analogue to the different timelines for development of girls and boys during postnatal life.

The present findings are not derived from extreme or pathologic cases but represent the healthy majority of pregnancies and point out that human development exhibit a measurable sensitivity to environmental factors already during the embryonic period. Note that the current yolk sac changes were associated with the sleep duration before pregnancy. Epigenetic programming has been linked both to sleep^9–17^ and immune progenitor cells produced in the yolk sac^59^. This opens the possibility of influencing and conditioning individual health development of the offspring right from the periconceptional period.

The present results made us also realize how rapid the pace of development is during the 1st trimester with sex and GA specific time windows that were susceptible to maternal and environmental effects (i.e., sleep and body composition). Future studies need to consider a correspondingly rapid frequency of observational windows to discern effects.

In addition, the two suggested explanatory models require further investigation; (a) the yolk sac compensation of a less favorable intrauterine environment due to a shorter prepregnant sleep duration and/or (b) an earlier ovulation or implantation due to a shorter prepregnant sleep duration. A precise determination of time of ovulation and implantation would be a good starting point^50^.

## Conclusion

Normal variation in prepregnant sleep does have an effect on embryonic development in healthy women; a shorter sleep duration corresponds to a larger yolk sac and CRL. Two features stand out; there is a sex-difference with an effect that appears to be exclusively among males, and the effect seems to operate during a short time window—around 7 weeks of pregnancy—underscoring the rapid pace of development that dominates the embryonic period.

### Supplementary Information

Supplementary Information 1.

Supplementary Information 2.

## Data Availability

All data generated or analysed during this study are included in this published article (and its supplementary information files).
