# Author Correction: Effect of maternal sleep on embryonic development

**DOI:** 10.1038/s41598-024-58405-z

**Published:** 2024-04-03

**Authors:** Alexander Vietheer, Torvid Kiserud, Øystein Ariansen Haaland, Rolv Terje Lie, Jörg Kessler

**Affiliations:** 1 Department of Obstetrics and Gynecology, Haukeland University Hospital, Jonas Lies Vei 72, 5053 Bergen, Norway; 2 Department of Clinical Science, Neonatal Research Group Western Norway, Maternal Fetal, University of Bergen, Bergen, Norway; 3 Department of Global Public Health and Primary Care, University of Bergen, Bergen, Norway; 4 Centre for Fertility and Health, Norwegian Institute of Public Health, Oslo, Norway

Correction to: *Scientific Reports* 10.1038/s41598-022-21516-6, published online 12 October 2022

The original version of this Article contained a typo in the Abstract.

“I.e., the yolk sac diameter decreased with increasing sleep duration (0.22 mm·h^−1^d^−1^, 95%CI [0.35–0.09], *P* < *0.01*), and CRL increased (0.92 mm·h^−1^d^−1^, 95%CI [1.77–0.08], *P* = 0.03).”

now reads:

“I.e., the yolk sac diameter decreased with increasing sleep duration (0.22 mm·h^−1^d^−1^, 95%CI [0.35–0.09], P < 0.01), and CRL decreased (0.92 mm·h^−1^d^−1^, 95%CI [1.77–0.08], P = 0.03).”

The original Article has been corrected.

