# Supplementary material for: Effect of maternal sleep on embryonic development
Source: Sci Rep. 2022 Oct 12;12:17099. doi: 10.1038/s41598-022-21516-6 (PMC9556600; doi:10.1038/s41598-022-21516-6)
Supplement: Supplementary file 1 — Supplementary Information 1. [file 41598_2022_21516_MOESM1_ESM.pdf]

Table S1 Prediction of the unstratified serial yolk sac measurements at gestational week 7 and 10 (in one group) by various sleep parameters: i.e., duration, difference between prepregnant and 1st trimester sleep duration (Δ-sleep), and sleep efficiency. Estimates calculated with mixed model linear regression accounting for gestational age—unstandardized regression coefficient (Effect); adjusted R squared (Adj.R2); 95% confidence interval (95%CI); AIC (Akaike information criterion). Two participants were excluded from the analysis due to uncertain information on LMP dates.

Term	*N*	Effect	95% CI	Adj.*r*2	AIC	*p*
**Yolk sac diameter by sleep duration before pregnancy ($mm \cdot h^{-1} \cdot d^{-1}$)**	172	-0.09	(-0.18– -0.00)	0.71	711.1	**0.04**
**Yolk sac diameter by sleep duration at end of 1st trimester ($mm \cdot h^{-1} \cdot d^{-1}$)**	174	-0.05	(-0.12–0.02)	0.71	718.6	0.14
**Yolk sac diameter by Δ-sleep ($mm \cdot h^{-1} \cdot d^{-1}$)**	169	0.02	(-0.06–0.10)	0.71	698.2	0.64
**Yolk sac diameter by sleep efficiency before pregnancy ($mm \cdot \%^{-1}$)**	173	-0.00	(-0.01–0.01)	0.71	715.6	0.86
**Yolk sac diameter by sleep efficiency at end of 1st trimester ($mm \cdot \%^{-1}$)**	174	0.00	(-0.01–0.01)	0.71	720.4	0.57

Table S2 Prediction of the unstratified (all) and sex-stratified serial crown-rump-length measurements in week 7, 10, and 13 (in one group) by total daily sleep duration before pregnancy and at gestational week 13. Estimated using mixed model regression accounting for gestational age—unstandardized regression coefficient (Effect); adjusted R squared (Adj.R2); 95% confidence interval (95%CI); AIC (Akaike information criterion).

Group	*N*	*Effect*	95% CI	Adj.*r*2	AIC	*p*
		**CRL by sleep duration before conception**				
**All**	174	-0.25 $mm \cdot h^{-1} \cdot d^{-1}$	(-1.17–0.66)	0.94	3386.0	0.58
**Male**	89	-0.56 $mm \cdot h^{-1} \cdot d^{-1}$	(-1.89–0.79)	0.92	1810.2	0.41
**Female**	84	0.10 $mm \cdot h^{-1} \cdot d^{-1}$	(-1.18–1.39)	0.95	1512.2	0.88
		**CRL by sleep duration at the end of the 1st trimester**				
**All**	176	-0.40 $mm \cdot h^{-1} \cdot d^{-1}$	(-1.17–0.28)	0.94	3410.5	0.23
**Male**	90	-0.20 $mm \cdot h^{-1} \cdot d^{-1}$	(-1.36–0.95)	0.92	1823.0	0.72
**Female**	85	-0.57 $mm \cdot h^{-1} \cdot d^{-1}$	(-1.53–0.38)	0.95	1520.8	0.23

Figure S1 First (week 7) and second (week 10) yolk sac measurements (in total N=358) presented as longitudinal observations by gestational age (here based on the fetal crown-rump-length). The individual pregnancies are distinguished by the color gradient.

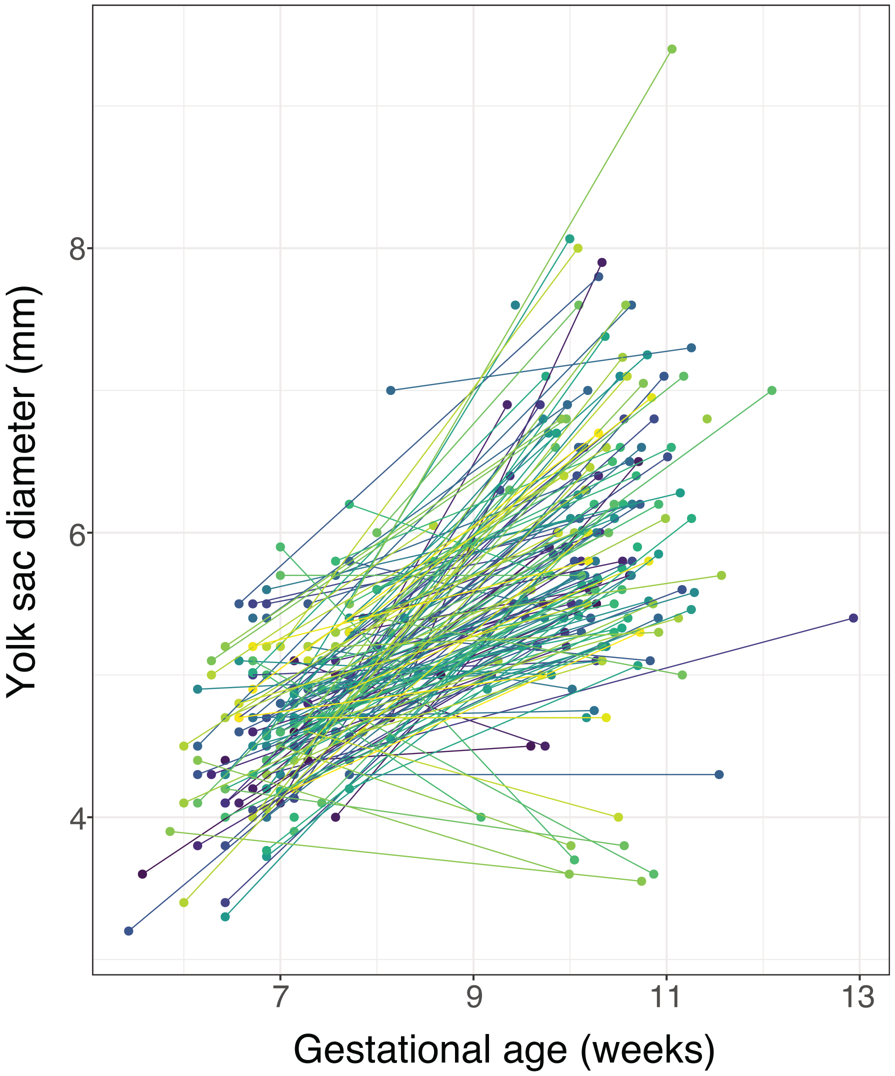

Table S3 Prediction of the yolk sac growth rate in mm/week (all, males, and females) between gestational week 7 and 10 by daily preconception sleep duration (in hours), sleep duration at end of first trimester, and their individual differences (end of 1st trimester–preconception). Calculated by ordinary least square regression models—unstandardized regression coefficient (Effect); adjusted R squared (Adj.R2); 95% confidence interval (95%CI); AIC (Akaike information criterion).

Group	*N*	Effect	95% CI	Adj.*r2*	AIC	*p*
		**Yolk sac growth rate by sleep duration before conception**				
**All**	156	0.002 mm/week·h^{-1}·d^{-1}	(-0.004–0.007)	-0.005	-619.5	0.60
**Male**	76	0.003 mm/week·h^{-1}·d^{-1}	(-0.004–0.01)	-0.004	-317.6	0.40
**Female**	79	-0.000 mm/week·h^{-1}·d^{-1}	(-0.008–0.008)	-0.012	-299.4	0.98
		**Yolk sac growth rate by sleep duration at the end of 1st trimester**				
**All**	159	0.000 mm/week·h^{-1}·d^{-1}	(-0.004–0.005)	-0.006	-640.3	0.85
**Male**	77	-0.001 mm/week·h^{-1}·d^{-1}	(-0.008–0.005)	-0.011	-322.1	0.66
**Female**	81	0.002 mm/week·h^{-1}·d^{-1}	(-0.005–0.008)	-0.009	-314.5	0.59
		**Yolk sac growth rate by Δ-sleep duration**				
**All**	153	0.000 mm/week·h^{-1}·d^{-1}	(-0.005–0.005)	-0.006	-617.6	0.90
**Male**	75	-0.004 mm/week·h^{-1}·d^{-1}	(-0.012–0.003)	0.007	-313.8	0.22
**Female**	77	0.004 mm/week·h^{-1}·d^{-1}	(-0.003–0.011)	0.001	-301.8	0.30

Figure S2 Unadjusted quantile regression-lines for the yolk sac size at the first measurement (week 7) by total daily preconception sleeping time in hours (*N*=165); median (thick black line) and 5th, 20th, 30th, 40th, 60th, 70th, 80th, and 95th percentile (grey); individual observations (open circles); ordinary least square regression-line (red).

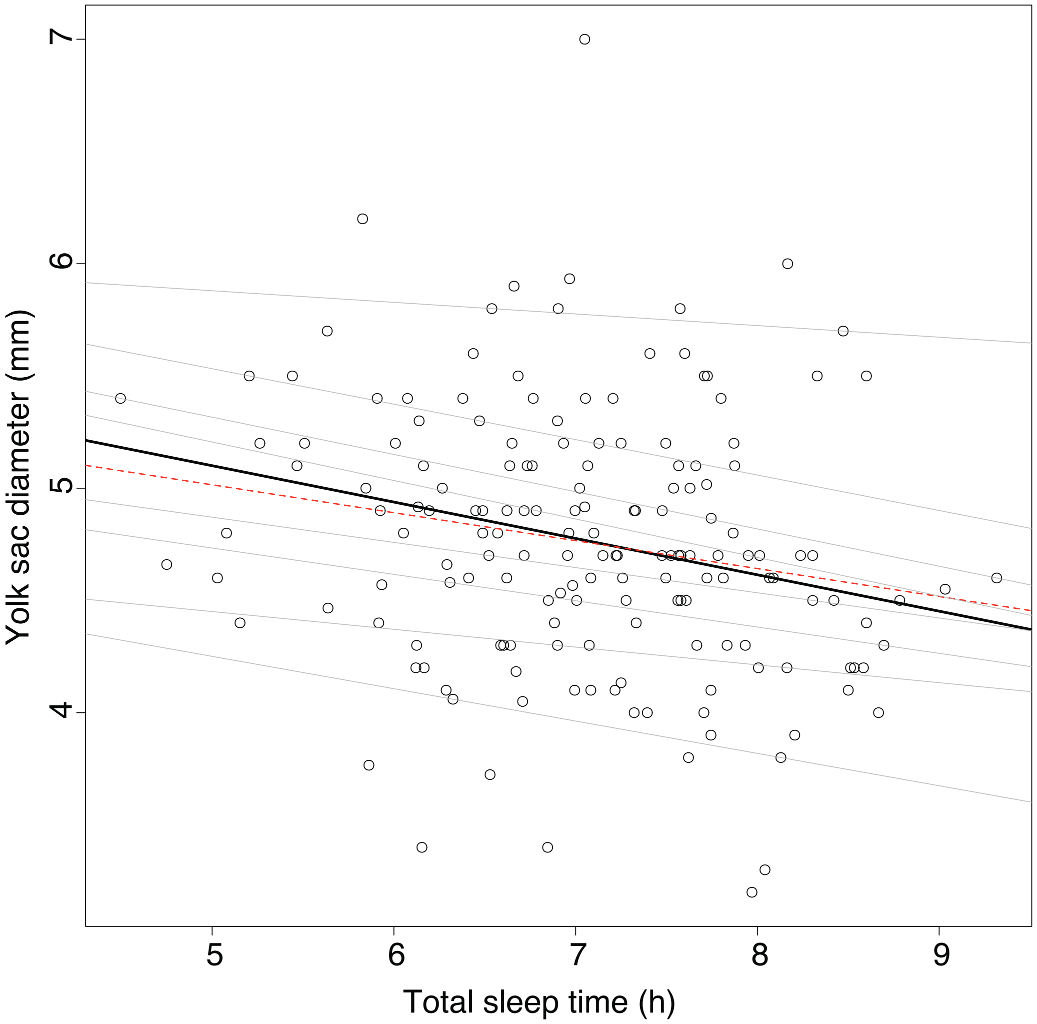

Figure S3 Overview of the results of the quantile regression models: The estimates on the y-axis with 95% confidence intervals (change of yolk sac size in mm per hour of daily sleep duration) were calculated for yolk sac deciles on the x-axis; zero-effect (red line)

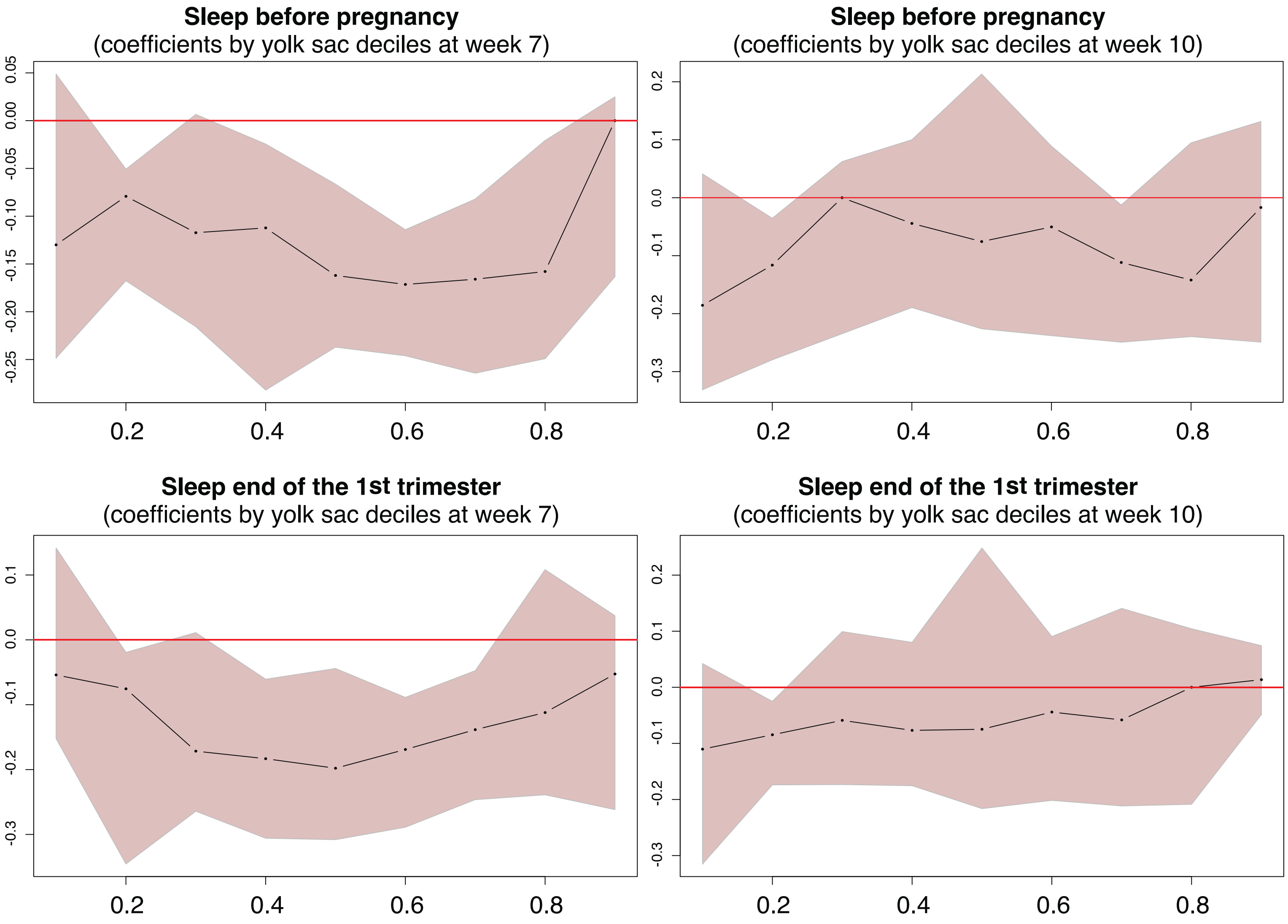

Table S4 Prediction of the yolk sac diameter (males and females) at gestational week 7 by total daily sleep duration before pregnancy. Calculated by ordinary least square regression models—unstandardized regression coefficient (Effect); adjusted R squared (Adj.R2); 95% confidence interval (95%CI); AIC (Akaike information criterion). The crude model (grey background) with its estimates and significance level can be compared with the adjusted models listened below, controlling for maternal health parameters: i.e., age, parity, physical activity level before conception (PA), height, weight, BMI, lean body mass (LBM), body fat percent (BFP), and gestational age (GA). The sleep-yolk sac relation remained significant after adjustments that caused minute changes in the estimated effect.

**Model**	***N***	**Effect**	**95% CI**	**Adj.*r*2**	**AIC**	***p***
**Crude**	165	-0.12 $mm \cdot h^{-1} \cdot d^{-1}$	(-0.22 to -0.03)	0.032	291.4	**0.01**
**Adjusted by age**	165	-0.12 $mm \cdot h^{-1} \cdot d^{-1}$	(-0.22 to -0.03)	0.026	293.4	**0.01**
**Adjusted by parity**	165	-0.12 $mm \cdot h^{-1} \cdot d^{-1}$	(-0.22 to -0.02)	0.047	296.6	**0.02**
**Adjusted by PA**	165	-0.12 $mm \cdot h^{-1} \cdot d^{-1}$	(-0.22 to -0.03)	0.022	295.9	**0.01**
**Adjusted by height**	165	-0.12 $mm \cdot h^{-1} \cdot d^{-1}$	(-0.22 to -0.03)	0.030	292.6	**0.01**
**Adjusted by weight**	165	-0.13 $mm \cdot h^{-1} \cdot d^{-1}$	(-0.22 to -0.03)	0.026	293.3	**0.01**
**Adjusted by BMI**	165	-0.11 $mm \cdot h^{-1} \cdot d^{-1}$	(-0.21 to -0.00)	0.027	293.2	**0.01**
**Adjusted by LBM**	165	-0.12 $mm \cdot h^{-1} \cdot d^{-1}$	(-0.22 to -0.02)	0.032	292.4	**0.01**
**Adjusted by BFP**	165	-0.12 $mm \cdot h^{-1} \cdot d^{-1}$	(-0.22 to -0.02)	0.017	293.1	**0.02**
**Adusted by GA**	164	-0.10 $mm \cdot h^{-1} \cdot d^{-1}$	(-0.19 to -0.01)	0.091	281.8	**0.03**

Table S5 Prediction of the yolk sac diameter (males and females) at gestational week 7 by total daily sleep duration before pregnancy. Calculated by ordinary least square regression models simultaneously adjusted for age, number of previous children, physical activity level before conception, height, lean body mass, body fat percent, and gestational age—unstandardized regression coefficient (Effect); adjusted R squared (Adj.R2); 95% confidence interval (95%CI); AIC (Akaike information criterion). The effect on male embryos remains significant.

**Group**	**N**	**Effect**	**95% CI**	**Adj.*r*2**	**AIC**	***p***
**All**	**165**	-0.08 $mm \cdot h^{-1} \cdot d^{-1}$	(-0.18 – 0.01)	0.056	297.7	0.09
**Male**	**83**	-0.19 $mm \cdot h^{-1} \cdot d^{-1}$	(-0.32 to -0.05)	0.16	138.7	**<0.01**
**Female**	**81**	-0.01 $mm \cdot h^{-1} \cdot d^{-1}$	(-0.15–0.16)	0.029	163.4	0.95

Table S6 Variable key variables table S7 (dataset)

Id	Study inclusion number
Age	Age of the participant at inclusion
para	Number of previous births
mens.cyc	Average length of menstrual cycle at inclusion
hypert.dis	Hypertensive disease or preeclampsia during the studied pregnancy
gdm	Gestational diabetes mellitus
wt.1	Weight at inclusion
ht	Height at inclusion
bmi.1	BMI at inclusion
bfp.1	Body fat percent at inclusion
lbm.1	Lean body mass at inclusion
physac	Level of weekly physical activity at inclusion
child.wt	Weight of the child at birth
apgar5	APGAR-score after 5 minutes
apgar10	APGAR-score after 10 minutes
childSex	Sex of the child
tot.preg.length.crl.mix	Total pregnancy length calculated from gestational age by earliest available fetal crown-rump-length
preterm	Preterm birth
lmp	First day of the last menstrual period
g_date.1	Date of the first assessment in pregnancy (week 7)
crl.1	Fetal crown-rump-length at the first assessment (week 7)
ys.1	Yolk sac diameter at the first assessment (week 7)
Examininer7	Identification number of the examiner at the first assessment (week 7)
g_date.2	Date of the second assessment in pregnancy (week 10)
crl.2	Fetal crown-rump-length at the second assessment (week 10)
ys.2	Yolk sac diameter at the second assessment (week 10)
Examininer10	Identification number of the examiner at the second assessment (week 10)
g_date.3	Date of the third assessment in pregnancy (week 13)
crl.3	Fetal crown-rump-length at the third assessment (week 13)
Examininer13	Identification number of the examiner at the third assessment (week 13)
sw.0.days.before.concept	Number of days from first actigraphy recording to ovulation (day 14 from LMP)
sw_date.1	Date of the first actigraphy recording (inclusion)
sw.ndays.1	Number of complete days (on body time≥94% per 24 hours) at the first recording
tst.1	Mean total sleep duration per day in minutes at the first recording
se.1	Sleep efficiency in % at the first recording
sw_date.2	Date of the second actigraphy recording (week 13)
sw.ndays.2	Number of complete days (on body time≥94% per 24 hours) at the second recording
tst.2	Mean total sleep duration per day in minutes at the second recording
se.2	Sleep efficiency in % at the second recording
excl	Exclusions: excluded 0; if excluded, reason by number 1–5
